# G3 and G9 Rotavirus genotypes in waste water circulation from two major metropolitan cities of Pakistan

**DOI:** 10.1038/s41598-020-65583-z

**Published:** 2020-05-26

**Authors:** Syeda Sumera Naqvi, Sundus Javed, Saadia Naseem, Asma Sadiq, Netasha Khan, Sadia Sattar, Naseer Ali Shah, Nazish Bostan

**Affiliations:** Department of Biosciences, COMSATS University, Islamabad, Pakistan

**Keywords:** Rotavirus, Viral reservoirs

## Abstract

Rotavirus A (RVA) is a diarrheal pathogen affecting children under age five, particularly in developing and underdeveloped regions of the world due to malnutrition, poor healthcare and hygienic conditions. Water and food contamination are found to be major sources of diarrheal outbreaks. Pakistan is one of the countries with high RVA related diarrhea burden but with insufficient surveillance system. The aim of this study was to gauge the RVA contamination of major open sewerage collecting streams and household water supplies in two major metropolitan cities of Pakistan. Three concentration methods were compared using RNA purity and concentration as parameters, and detection efficiency of the selected method was estimated. Water samples were collected from 21 sites in Islamabad and Rawalpindi in two phases during the year 2014–2015. Meteorological conditions were recorded for each sampling day and site from Pakistan Meteorological Department (PMD). Nested PCR was used to detect the presence of RVA in samples targeting the VP7 gene. Logistic regression was applied to assess the association of weather conditions with RVA persistence in water bodies. Statistical analysis hinted at a temporal and seasonal pattern of RVA detection in water. Phylogenetic analysis of selected isolates showed a close association of environmental strains with clinical RVA isolates from hospitalized children with acute diarrhea during the same period. This is the first scientific report cataloging the circulating RVA strains in environmental samples from the region. The study highlights the hazards of releasing untreated sewerage containing potentially infectious viral particles into collecting streams, which could become a reservoir of multiple pathogens and a risk to exposed communities. Moreover, routine testing of these water bodies can present an effective surveillance system of circulating viral strains in the population.

## Introduction

Rotavirus A (RVA) is an important diarrhea causing pathogen responsible for high morbidity and mortality rates in developing and underdeveloped regions of Africa and Asia. An estimated 215,000^1^ children aged less than 5 years die from rotavirus associated diarrhea every year, with more than 85% of these deaths occurring in low-income countries^2,3^. This triple layered, 70 nm, virus enters host via oral-fecal route. Dehydration is the main complication leading to death in RVA diarrhea^4^. This risk is especially high in malnourished and poor children who do not have timely access to medical facilities^5^. RVA disease seasonality shows that cold dry season supports the survival and transmission of rotavirus^6^.

Water, food and fomites contaminated with fecal material are important vehicles for RVA transmission. It is estimated that as many as 10^11^ particles of RVA are shed per gram of fecal material, while the viral infectious dose is 10–100 virus particles^7^. RVA can survive on human hands for four hours and can persist in air and on fomites in relatively less humidity^8^. RVA has been shown to tolerate a wide range of physical conditions like free chlorine^9^, pH (3–10), temperature, humidity and chemical disinfection^10^. The virus adsorbs to particulate matter in water that enhances its survival. Due to this remarkable stability and persistence, it is not surprising that the virus has been isolated from wastewater, fresh water, ground water, drinking water^11,12^, shellfish and vegetables harvested from contaminated water as well as soil^13^.

Pakistan is a country with about 20 million people with 15% of population under age five. The country has high rotavirus associated morbidity with 0.14 million cases per annum, some leading to mortality. In year 2000, 5.6% death rate due to RVA infection was estimated^14^. Hospital based studies conducted in previous years have determined that RVA is responsible for about one third of children with severe diarrhea especially below 2 years of age. The incidence rate was found to be about 5.7–8.1 per thousand children^15^.

An extensive system of natural streams known as Nullah Lai and its tributaries flow through Rawalpindi and Islamabad (capital city). It has a catchment area of 234.8 km^2^ and stretches almost 30 km from the foothills of Margalla to Soan river, receiving drainage and waste matter from twin cities (Rawalpindi and Islamabad) along the course and finally joins Soan and Korang River respectively^16,17^. A number of reports have described RVA associated diarrheal outbreaks in this region but data regarding high risk populations is missing. The remarkable persistence of rotavirus in water makes this natural stream system used by twin cities for sewerage drainage an excellent resource of RVA surveillance. In our knowledge, this is the first study in Pakistan designed to detect RVA in the surface water samples from Rawalpindi and Islamabad.

## Results

### RVA concentration

Detection of viral particles from water samples is a challenge due to low viral load. Therefore, different viral concentration methods were used prior to viral detection. Criteria used for evaluation of concentration methods in this study were RNA concentration and purity, data is shown in Table 1 and RNA degradation (data not shown). Method I yielded highest RNA concentration 1375(SD = 31.9) ng/µl and RNA purity was 1.41 (SD = 0.072) (RNA purity >1.6 is suitable for molecular studies, while ideal ratio is 2.0). The RNA purity in concentrates generated by method II was 1.55 (SD = 0.058), while the concentration was 237.3 (SD = 46.7) (acceptable is >200 ng/µl). Maximum RNA purity was achieved using method III 1.94 (SD = 0.07) and RNA yield was 530.7 (SD = 15.5). The high yield of RNA in method I was probably due to presence of contamination at A_260_ (data not shown) that may have led to overestimation.

**Table 1:** Comparison of viral RNA yield and quality to determine the efficiency of three methods in concentrating Rotavirus.

	RNA Purity (260/280) Mean (SD)	RNA concentration (ng/µl) Mean (SD)
Method I	1.41 (0.072)	1375 (31.9)
Method II	1.55 (0.058)	237.3 (46.7)
Method III	1.94 (0.07)	530.7(15.5)

**Figure 1:**
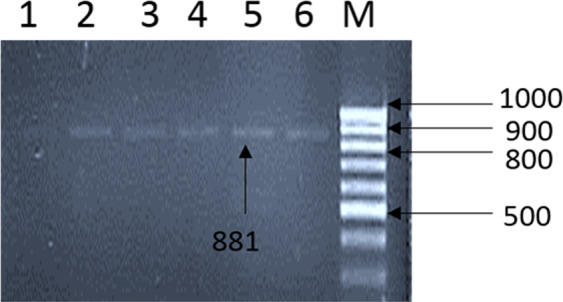
Agarose gel (1.5%) electrophoresis of nested PCR amplified product for VP7 gene of rotavirus (cropped). M (marker), 100 bp DNA ladder; Lane 1 to Lane 6, 10^−10^ 10^−8^ 10^−6^ 10^−4^ 10^−2^ 10^−0^ serial dilutions of stool sample containing positive sample (G1P^8^). (Original gel picture is attached in Supplementary Materials).

Meanwhile, to establish the detection limit of the selected method, serial dilutions of a RV positive stool sample were prepared. The 10% dilution of original stool sample was tested positive for rotavirus by ProspecT Rotavirus Microplate assay (R240396) with OD value 0.769 (Supplementary Figure I). According to manufacturer’s manual, a sample showing OD value of 0.76 contains 7.8 × 10^5^ virus particles/ml. The detection limit of Method III was estimated to be at 10^–8^ dilution concentration (Fig. 1).

### RVA prevalence in sewage water

During the first phase of sample collection, thirteen water samples from Islamabad and seven from Rawalpindi were collected. During this phase of sampling, 45% water samples collected from 10 sites were found positive for RVA. Five samples from Nullah Lai, three from its tributaries from Rawalpindi and Islamabad tested positive for RVA, while samples from Rawal dam and Banni gala were also positive for RVA. During the second round of sampling, RVA was not detected in any sample using same methods (Supplementary Table I). Overall detection rate of RVA in water during the whole study period was 23%.

### Analysis of climatic parameters associated with RVA detection

Incidence of RVA diarrhea shows seasonal patterns globally. It has a direct and immediate relationship with cold weather in different zones of world^18,19^. Although in Pakistan RVA diarrheal cases are reported throughout the year; incidence rate shows significant relationship to seasonality^20^. In this study, we investigated the effect of climatic factors on persistence of RVA in water. Statistical analyses revealed that climate conditions significantly impacted the presence of Rotavirus in water samples. Mann Whitney U test was applied to non-normally distributed data that showed that the environmental factors are significantly different between the two study groups. Relative humidity (*p* = *0.005*) and temperature (*p* = *0.005*), solar radiation (*p* = 0.005) wind speed (*p* = 0.02) seemed significantly different among the groups (Table 2). High relative humidity, low temperature, less solar radiation and slower wind speed were found associated with rotavirus presence in water samples. Univariate logistic regression, showed that temperature (OR = 0.72(95%CI = 0.50–0.88), *p* = 0.02) and solar radiation (OR = 0.70(95%CI = 0.49–0.87), *p* = 0.009) are negatively associated with RV presence. Moreover, relative humidity had a causal effect (OR = 1.27(CI = 1.08–1.64), *p* = 0.02) (Table 3). However, precipitation, pH of water and wind speed was not found significantly associated with RVA presence in water. In multivariable logistic regression analyses, temperature was adjusted for humidity and solar radiation, depicting temperature as the only co-dependent variable with RV presence in water (OR = 0.10(95%CI = 0.01–0.54), *p* = 0.02) (Table 3).

**Table 2:** Descriptive analysis of association of variables with Rotavirus presence and absence in water samples.

Samples	Mean (SD)/N (%)	Mean (SD)/N (%)	*p
	Negative	Positive	
Total (N = 42)	32 (74.4)	10(23.2)	
Average Temperature °C	22.03(7.02)	13.1(1.79)	0.001
Average Relative humidity%	58.72(8.18)	66.70(1.42)	0.005
Average Precipitation mm	61.48(70.1)	27.18(17.64)	0.67
Solar radiation mj/m^2^/day	16.94(5.0)	10.70(1.34)	0.005
Wind speed km/hour	4.22(2.2)	2.59(1.36)	0.02
pH of water	7.62(0.59)	7.19(0.19)	0.05

*p = Man whitteny U test p-values; significant values were <0.05.

**Table 3:** Logistic Regression Analysis showing association of weather variables with Rotavirus presence in water.

Univariable Analysis			*p*-value	Multivariable Analysis		*p*- value
Variables	OR	95% CI		AOR	95% CI	
Temperature	0.72	0.50–0.88	0.02	0.1	0.01–0.54	0.0222
Relative humidity	1.27	1.08–1.64	0.02	0.47	0.17–1.6	0.1063
Solar radiation	0.7	0.49–0.87	0.009	5.3	0.75–200	0.1655
Precipitation	0.99	0.96–1.00	0.17			
Wind speed	0.63	0.36–0.94	0.05			
pH of water	0.01	0.006–0.67	0.05			

OR = Odds Ratio, CI = Confidence Interval, AOR = Adjusted Odds Ratio;

significant values were <0.05.

### RVA genotypes in waste water

**Figure 2:**
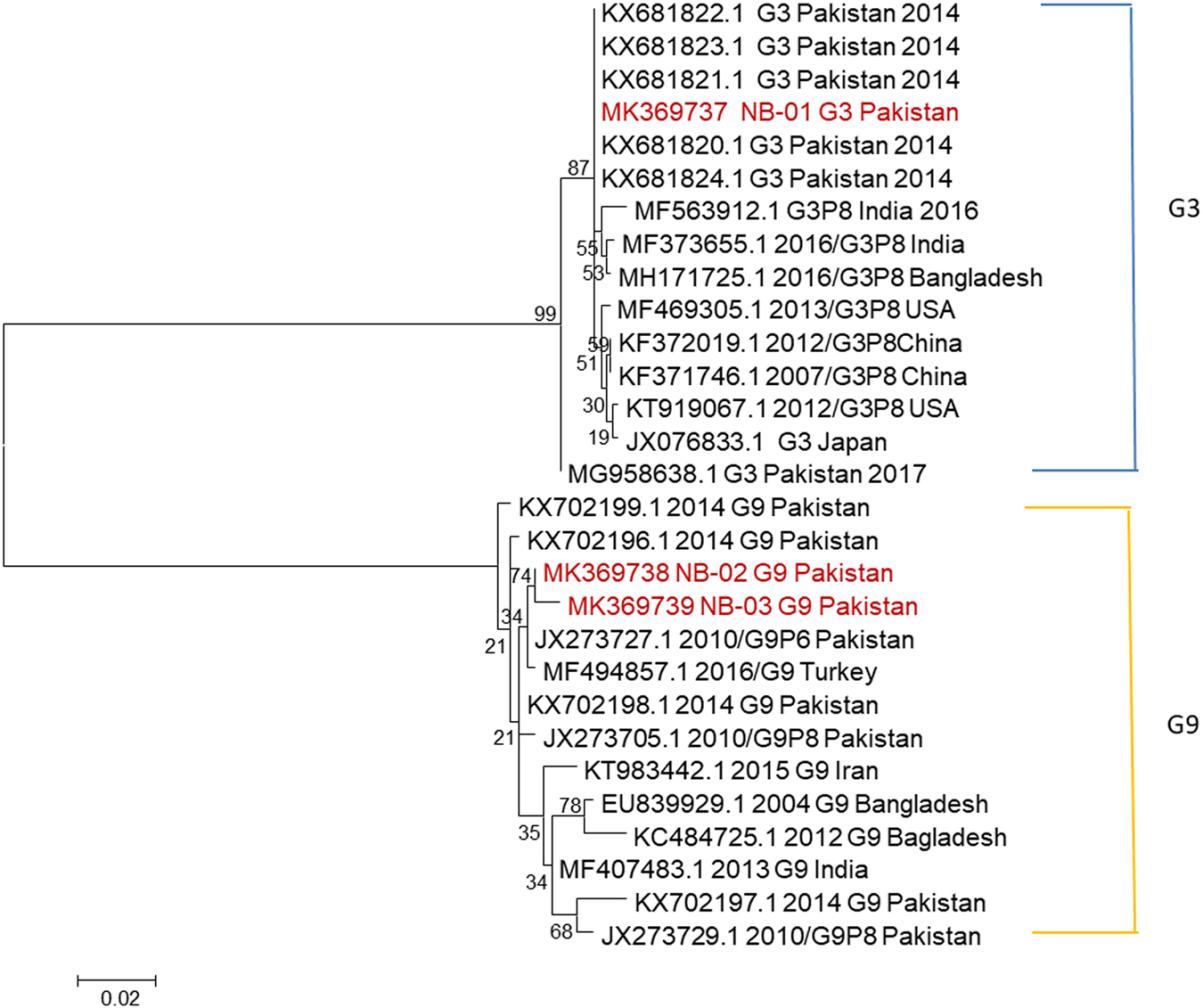
Maximum Likelihood Phylogenetic Tree of two G9 and one G3 strains of Rotavirus A (RVA) detected in this study, constructed in MEGA program 6.0 with kimura-2-parameter model using 1000 bootstrap replicates. The sequences generated in this study are highlighted in red color.

G3 and G9 genotypes were detected in waste water sample. Maximum likelihood tree was constructed for RVA genotypes based on partial sequencing of gene segment VP7 (Fig. 2). Phylogenetic analysis of Pakistani G3 strain sequenced in this study (MK369737) showed a 99.8 to 100 percent nucleotide identity with clinical G3 strains accession number KX681821, KX681822, KX681823, MH279580, MH279581, MH279583, MH279585 isolated in previously reported studies from Pakistan during the year 2014 and 2015^20,21^ as well as with strains detected in India and Bangladesh. Two environmental waste water G9 strains (MK369738 and MK369739) sequenced in current study showed more than >99% nucleotide identity with each other and had close association with strains detected in hospital patients infected with RVA from Pakistan during 2010 accession number JX273705 and 2014–2016 accession number KX702196, KX702198, KX702199, MH277395 and MH277396^20,21^.

## Discussion

RVA diarrhea is considered to be the main cause of childhood mortality particularly in developing countries like Pakistan. According to an estimate in 2013, Pakistan is one among four countries in the world with approximately half (45%) of deaths due to RVA diarrhea^22^. Water and food contamination is the major source of diarrheal spread. With the support of GAVI, the Vaccine Alliance, UNICEF and WHO the government of Pakistan has recently implemented RVA vaccination in the EPI schedule of each of the four provinces (Punjab, Khyber-Pakhtunkhwa, Sindh and Baluchistan) of the country^23^. The disease burden due to RVA diarrhea is expected to be reduced in the future after vaccine implementation. However, there are many additional challenges involved in improving health facilities and socio-economic conditions of houses in highly populated areas. Moreover, introduction of a vaccine imposes a shift in circulating viral strains^24^. Therefore, continued surveillance of viral genotypes in circulation is necessary for effective control.

Present study investigated circulating RVA strains in Nullah Lai and Rawal lake from twin cities (Rawalpindi/Islamabad) during 2014 and 2015. Although surveillance model is in place for detection of Polio virus in water bodies, not enough evidence is available of RVA circulation in water bodies of Pakistan. The meteorological risk factors associated with RVA persistence in water were also investigated. The overall prevalence rate of RVA in water during study period was 23%. The RVA detection rate in the present study is lower than the previous study from Karachi, which detected RVA in 35% of water filtration plants and 5% tap water using adsorption-elution virus concentration method with positively charged membranes^25^. On the other hand, an another study from Peshawar detected RVA in 9.47% of drinking and sewage water samples using adsorption-elution virus concentration method with negatively charged membranes^26^. Both studies used concentration methods that are expensive and costly in terms of implementation for routine surveillance purposes.

The concentration methods used in this study are relatively inexpensive and less laborious that can be widely employed in a developing country like Pakistan. A number of concentration methods were evaluated for viral RNA yield and quality that can be used for detection of RVA using PCR amplification. Method III using 10% PEG for virus concentration along with TRIzol Method for RNA extraction was able to detect virus particles at 10^–8^dilution. Using this concentration method along with downstream processing, overall 23% of water samples were tested positive for RVA.

A seasonal trend was observed as water samples collected during winter and autumn months tested positive for RVA. It is in contrast to studies that reported persistence of RVA throughout the year in some other parts of the world including tropical countries^27^, but in accordance with studies that reported temporal trends in persistence of RVA in water with more RVA detection in winter and autumn months^28^.

Rotaviruses are a large genetically diverse population which varies in each country^20^. The first Rotavirus outbreak from Pakistan was reported during 1983–1985. Over the years, the RVA incidence and genotype diversity has changed (Table 4). The most common G and P-genotypes affecting humans are G1, G2, G3, G4, G9, G12 and P[4], P[6] and P[8], respectively^29^. Previous studies from Pakistan have reported high prevalence of G and P genotypes including G1, G2, G3, G9, and P[4], P[6] and P[8], respectively^20^. In the present study two RVA genotypes (G3 and G9) were detected in water sample from Dhoke Naju locality in Rawalpindi. Sequence analysis of these strains indicated a close association (>99% identity) with RVA strains accession number KX 681821,KX681822, KX681823, MH279580, MH279581, MH279583, MH279585 detected in patients suffering from acute diarrheal illness in Rawalpindi during 2014–2016^20,21^, the same period as our study. G3 genotype has not previously been reported in Pakistan (Table 4). It was first detected during the year 2014–2015 and is now second most common genotype in patients suffering from RVA related acute diarrhea^20,21,30^. Similarly, our environmental G9 strains have high similarity with strains accession number KX702196, KX702198, KX702199, MH277395, MH277396 previously reported in Pakistan during 2014–2016^20,21,30^.

**Table 4:** Previous studies of RVA related gastroenteritis reported from Pakistan.

Study year	RV detection %age	Genotypes detected	Study area	Reference
1983–1985	9.6%	Nil	Rawalpindi	^40^
1993		G2	Pakistan	^41^
1990&1991	12.3%&24.4%		Karachi	^42^
1990 to 1997	13.70%	G1, G4, G2	Karachi	^43^
2008		G12	Rawalpindi	^44^
2005–2007	57%	G1, G2, G9, G3	Faisalabad	^45^
2010	23.80%	G1, G2, G6, G9, G12	Rawalpindi	^30^
2008–2009	34%	G1, G2, G9	Lahore	^46^
2006–2008	30.50%	G1, G2, G9, G4	Karachi, Lahore, Rawalpindi, and Peshawar	^47^
2014	29%	G1, G2, G3, G9, G12	Rawalpindi	^21^
2015–2016	26.8%	G1, G2, G3, G9, G12	Rawalpindi	^20^

Nullah Lai basin is prone to flooding as happened in case of unprecedented rainfalls like in 2001. Muhallah Raja sultan and Dhoke Naju were badly affected by flooding^31^, while Rawal lake is a recreation point and source of water supply to Rawalpindi and Islamabad households. Frequently children take a swim in these waters. RVA is a highly contagious virus and its persistence in the water bodies can pose a serious public health threat in settings with poor sanitation and hygienic conditions and in less efficient water treatment plants^32^. During this study, water sample from Dhoke Naju was used for sequencing for RVA strains. Dhoke Naju locality is present along one of major converging points of streams in almost plain area of Nullah Lai basin. Nullah Lai may not be a direct environmental hazard to communities living along banks, but still can pose threat if this water is used for agriculture and bathing animals, as is frequently observed. Also, it could be an excellent source for monitoring changing genotypic diversity of rotavirus strains in twin cities that can advise vaccination campaigns. Although this study has constrains of short study period, small sample size and inclusion of only two sentinel sites, it is promising and can be used for disease surveillance on large scale. The above mentioned limitations should be considered for better understanding of disease surveillance in future studies.

This study concludes the first ever reported prevalence of G3 and G9 RVA genotypes in water bodies in Pakistan. It highlights the close association of environmental RVA strains with disease associated clinical isolates reported from hospitals during the same period. Shedding of viral particles in excreta in sewage water can be significant in determining the circulating genotypes prevalent in a community rather than investing huge funding in population surveillance. Moreover, it is an inert way to get the complete picture of circulating viral types in the community by indirect sampling and without involving patients/population at risk and community etc. These types of studies are highly advisable for enteric viruses’ surveillance utilizing waste water bodies.

## Methods

### Sampling sites

**Figure 3:**
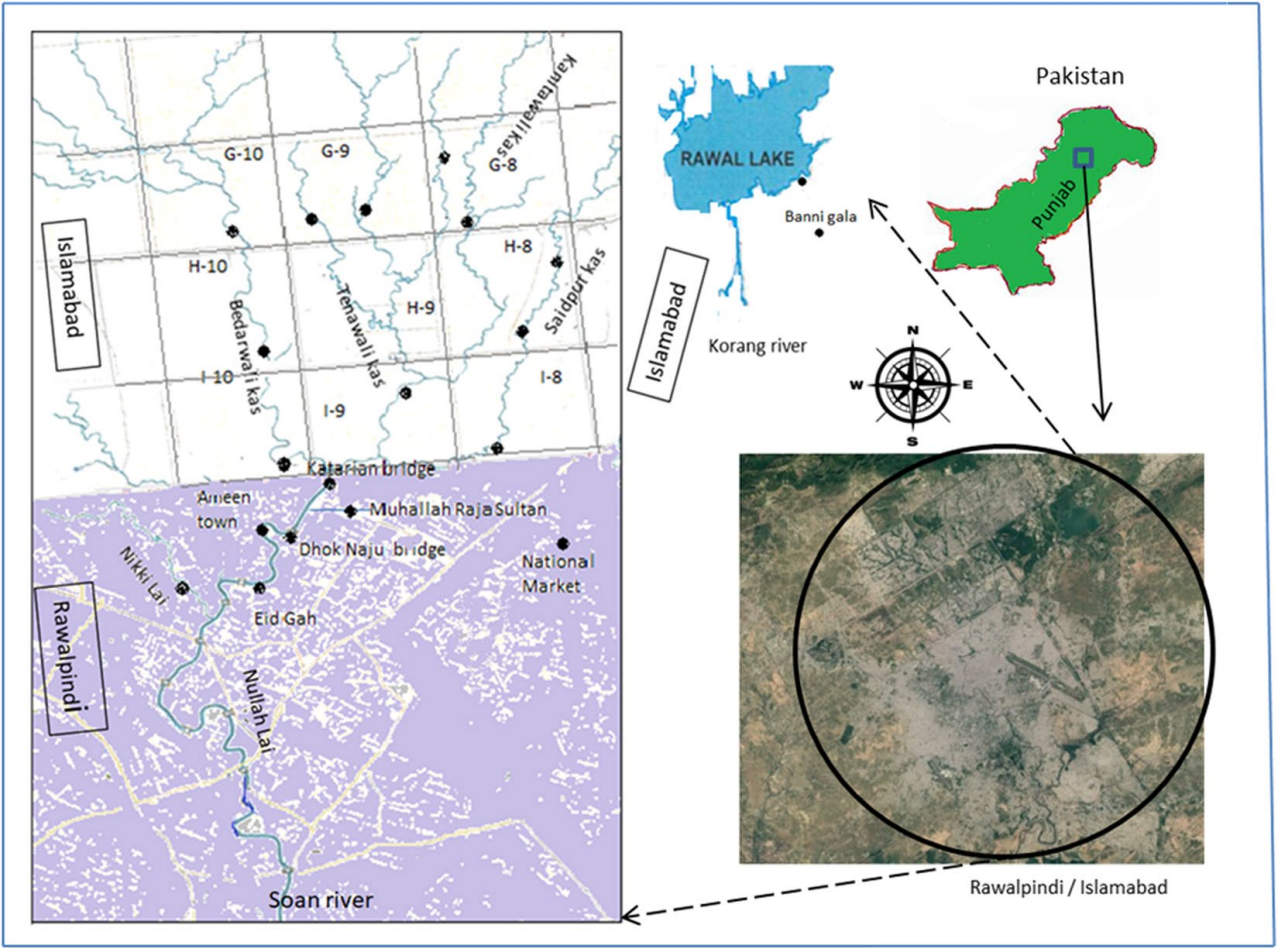
Map showing sampling sites in twin cities of Islamabad (Capital city of Pakistan) and Rawalpindi. Imagery taken from Google Earth: Image 2018 © DigitalGlobe and annotated using Microsoft © 2018 built in apps.

The three main tributaries of Nullah Lai pass through less populated and also residential sectors of Islamabad, and conjoin as main Nullah Lai before entering into densely populated areas of Rawalpindi. The sampling sites were selected based on ease of access, proximity to drainage points and conjoining points of tributaries. For this study, twenty sites along Nullah Lai and its tributaries and one site along Korang river tributary and one from Rawal dam was selected (Fig. 3).

### Sample collection

In total, 42 samples were collected. First phase of sampling was conducted from November 2014 to February 2015 and second phase of sampling was undertaken during the months of April 2015 to July 2015. One liter water was collected in duplicate, along the bank of Nullah, in clean, sturdy and clear bottles using Grab sampling method. Samples were transported in cold conditions to Molecular Virology laboratory at COMSATS University Islamabad campus and were stored at 4 °C.

### Meteorological conditions

Meteorological data (temperature, relative humidity, precipitation, solar radiation, wind speed) for each sampling day was obtained from Pakistan Metrological Department (PMD) and pH of each sample was measured (Mi 150). Samples were further processed within 48 hours for RVA detection.

### Virus concentration

Initially, three concentration methods were taken from literature to adopt a suitable method for this study. For this purpose, a surface water sample collected from Muhalla Raja Sultan locality (Supplementary Table I) was concentrated by these methods and their efficiency was determined by comparing the extracted RNA purity and concentration yield using a Nanodrop spectrophotometer (Implen Nanophotometer-Germany).

#### Method I

Pellet method: Concentration method of Kargar and coworkers was used^33^, where 100 ml of sewage water was centrifuged at 4000 rpm for 30 minutes at 5 °C. Tubes were kept cold in ice bucket and upper aqueous phase was removed until 4 ml of pellet solution was left. 1 ml of pure chloroform was added to this and pellet was dissolved by vigorous shaking. The tubes were centrifuged first at 200 × g for 20 minutes, then at 2000 × g for 10 minutes. Upper aqueous phase was shifted to a sterile tube and stored at −20 °C.

#### Method II

500 ml of water sample was centrifuged at 1000 × g for 30 minutes. Supernatant I was removed and kept at 4 °C, while pellet was dissolved in 10 mL of supernatant I. 1 mL of chloroform was then added and centrifuged at 1000 × g for 5 minutes. Supernatant II was removed and combined with supernatant I. Then 0.2 M NaCl and 8% PEG 6000 (Fluka, Germany) were added to the total volume respectively, stored at 4 °C overnight and centrifuged at 2000 × g for 2 hours at 4 °C. Resulting pellet was resuspended in PBS and stored at −20 °C^34,35^.

#### Method III

This was a modified version of method II. Briefly, 500 mL of water sample was centrifuged at 4000 × g for 30 minutes. 0.5 M NaCl (Sigma) and 10% PEG 6000 (Fluka, Germany) was added to supernatant, respectively and stirred for 30 minutes at 4 °C with magnetic stirrer. After overnight incubation at 4 °C, the sample was centrifuged for 35 minutes at 7000 rpm at 4 °C (Backman model J2_21 centrifuge). Supernatant was drained and pellet was resuspended in 10 ml supernatant and mixed with 1 ml of pure chloroform and centrifuged at 2000 × g for 10 minutes at 4 °C. Pellet was discarded and PEG precipitation step (same as above) was repeated with the supernatant. The final pellet containing virus was dissolved in 1 mL of PBS.

### RNA extraction

Total RNA was extracted from 250 µl of concentrated water sample using 750 µl TRIzol LS (Invitrogen) according to established protocol^36^. RNA was eluted in 20 µl nuclease free water and stored at −20 °C.

### Determination of detection limit of method III

The detection limit of the selected method, out of three, was then estimated by preparing suspension of two grams of Rotavirus positive fecal sample in 10 ml autoclaved distilled water. This dilution of original stool sample was considered 10° dilutions in this series and was then used to prepare twofold serial dilutions upto10^−10^ dilutions.

### Reverse transcriptase polymerase chain reaction (RT-PCR)

Purified RNA (5 µl) was reverse transcribed by using a 20 µl reaction mix of primers Beg 9 and End 9 (10 pMol/µl) (Supplementary Table II), 1 × RT buffer, 1mM dNTPs, 40 U of RNase inhibitor, 200 U of reverse transcriptase enzyme^33,37^. The reaction was first heated at 37 °C for 5 minutes, then 48 °C for 60 minutes and lastly 5 minutes at 95 °C. The concentration of cDNA was measured using Nanodrop spectrophotometer (Implen Nanophotometer-Germany) and reduced to final concentration of 50 ng/µl.

### VP7 gene amplification

VP7 gene of Rotavirus was amplified through Nested PCR. A reaction volume of 25 µl containing 1–2 µl cDNA, Beg 9 and End 9 primers (10 pMol/ul), 1 × PCR buffer, 0.5 mM dNTPs, 1.2 mM MgCl_2_ and 1 U Taq polymerase enzyme was used. Amplification was carried out at following thermal cycling parameters: 95 °C for 5 minutes, 40 cycles of 94 °C for 30 sec, 48 °C for 45 sec, 72 °C for 1 min and final extension at 72 °C for 10 min^37^. Second round of Nested PCR was performed in 25 µl reaction volume containing 1st round PCR product, VP7F and VP7R primers (Supplementary Table II). Cycling parameters used were: 95 °C for 5 minutes. 40 cycles of 94 °C for 30 sec, 50 °C for 45 sec, 72 °C for 1 min. Final extension was performed at 72 °C for 10 min.

### Statistical analysis

All statistical analyses were performed using R version 3.2.2. Environmental variables, such as temperature, relative humidity, precipitation, solar radiation and wind speed were compared using non-parametric Mann-Whitney U-test. Association of weather conditions with detection of rotavirus in water samples (positive verses negative) was tested using logistic regression analysis. The results were then adjusted for temperature, relative humidity and solar radiation. The nominal threshold for significance was considered as <0.05.

### Sequence analysis

VP7 PCR product of RVA positive sample was excised and purified using the ExoSAP clean-up kit (Thermofisher Scientific, USA) to remove excess nucleotides and primers. The PCR amplicons were then sequenced using the BigDye® Terminator Cycle Sequencing Kit (Life Technologies, USA). Sequencing was performed on cDNA with the forward primer VP7F. Following the sequencing, the reactions were cleaned up by performing ethanol precipitation and the final product was loaded in automated DNA Analyzer ABI 3100 (Applied Biosynthesis).

### Determination of RVA genotypes

VP7 sequences were assembled using SeqMan Pro (Lasergene package, DNAstar Inc., Madison, Wisconsin, USA). The sequences were manually corrected and compared with referential strains available online in GenBank using Nucleotide BLAST tool of NCBI. The genotypes were determined by using RotaC 2.0 v (http://rotac.regatools.be/), the RVA online classification tool^38^. Sequences were submitted to GenBank and were assigned the following accession numbers MK369737, MK369738 and MK369739.

### Phylogenetic analysis

Sequences were aligned using ClustalW and phylogenetic trees were generated in MEGA 6.06 by Maximum Likelihood method with kimura-2-parameter model using 1000 bootstrap replicates for statistical reliability^39^.

## Supplementary information

G3 and G9 Rotavirus genotypes in waste water circulation from two major metropolitan cities of Pakistan.

## Data Availability

The authors declare that they will share all available data as per required by the referees or the editorial office.
