# Supplementary material for: G3 and G9 Rotavirus genotypes in waste water circulation from two major metropolitan cities of Pakistan
Source: Sci Rep. 2020 May 26;10:8665. doi: 10.1038/s41598-020-65583-z (PMC7251132; doi:10.1038/s41598-020-65583-z)
Supplement: Supplementary file 1 — G3 and G9 Rotavirus genotypes in waste water circulation from two major metropolitan cities of Pakistan. [file 41598_2020_65583_MOESM1_ESM.docx]

**G3 and G9 Rotavirus genotypes in waste water circulation from two major metropolitan cities of Pakistan**

**Authors:**

Syeda Sumera Naqvi^1^, Sundus Javed^1^, Saadia Naseem^1^, Asma Sadiq^1^, Netasha Khan^1^, Sadia Sattar^1^, Naseer Ali Shah^1^ and Nazish Bostan^1^*

**Author affiliations:**

^1^Department of Biosciences, COMSATS University, Islamabad, Pakistan

**Corresponding Authors:**

^*^ Dr. NazishBostan

Phone No. +92 51 9049 6102

Email: [nazishbostan@comsats.edu.pk](mailto:nazishbostan@comsats.edu.pk)

**Supplementary figure I:** ELISA results of suspected Rotavirus stool samples tested by ProspecT Rotavirus Microplate assay (R240396). E1 shows the O.D value for stool sample used for preparing dilution series. According to manufacturer’s manual, a sample showing OD value of 0.76 contains 7.8 x 10^5^ virus particles/ml. A1 is negative control and B1 is positive control.

**Kit manual:**

**
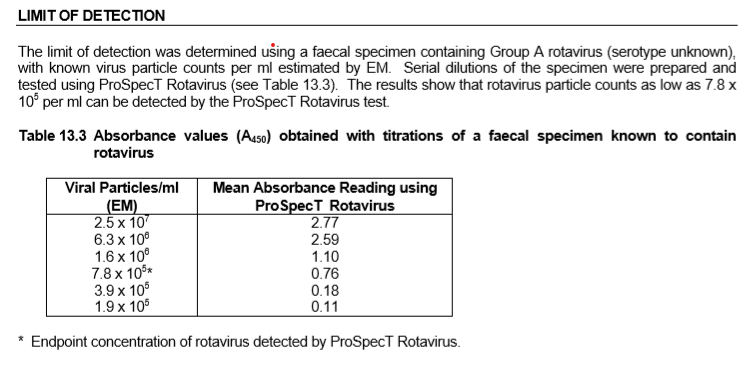
**

**Supplementary figure II:** Original gel picture

**
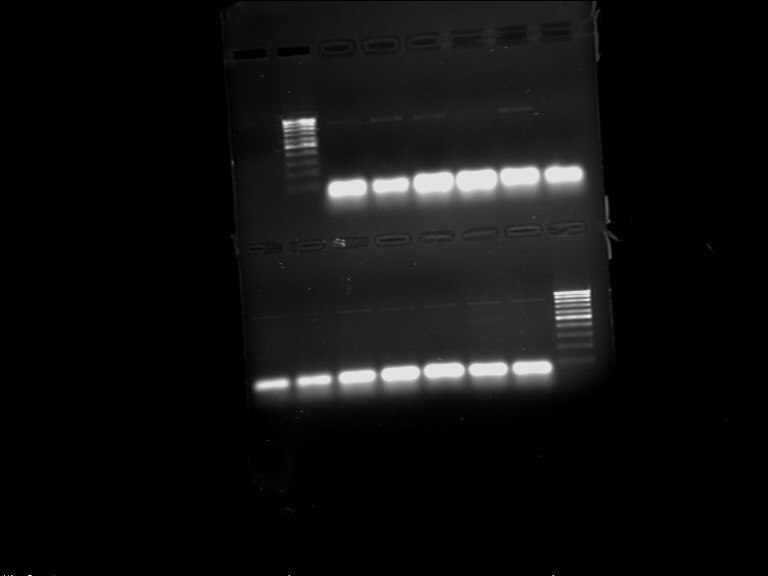
**

**Supplementary table 1:** Weather condition during both phases of study period

| **Area** | **Month** | **Temperature (average)0 C** | **Relative Humidity (average)** | **Monthly Precipitation mm** | **Solar Radiation mj/m2/day** | **Wind Speed km/h** | **Rotavirus detection** | **pH of water** |
| --- | --- | --- | --- | --- | --- | --- | --- | --- |
| G-10/4 | Nov-14 | 16 | 65 | 5.8 | 13 | 0.5 | negative | 6.98 |
| G-9/1 | Nov-14 | 16 | 65 | 5.8 | 13 | 0.5 | negative | 7.13 |
| G-9/4 | Nov-14 | 16 | 65 | 5.8 | 13 | 0.5 | negative | 7.08 |
| G-8/2 | Nov-14 | 16 | 65 | 5.8 | 13 | 0.5 | positive | 7.25 |
| G-8/1 | Nov-14 | 16 | 65 | 5.8 | 13 | 0.5 | positive | 7 |
| H-10 | Dec-14 | 12 | 70 | 0.2 | 9 | 3.4 | negative | 7.04 |
| H-9/4 | Dec-14 | 12 | 70 | 0.2 | 9 | 3.4 | negative | 7.24 |
| H-8/4 | Dec-14 | 12 | 70 | 0.2 | 9 | 3.4 | negative | 7.2 |
| H-8/1 | Dec-14 | 12 | 70 | 0.2 | 9 | 3.4 | negative | 7.3 |
| I-10/4 | Jan-15 | 12 | 67 | 32 | 10 | 2.5 | negative | 6.82 |
| I-8/1 | Dec-14 | 12 | 70 | 0.2 | 9 | 3.4 | negative | 7.17 |
| I-9 | Dec-14 | 11 | 70 | 0.2 | 9 | 3.4 | positive | 7.1 |
| New katarian bridge | Jan-15 | 12 | 67 | 32 | 10 | 2.5 | positive | 7.15 |
| Ameen Town | Jan-15 | 12 | 67 | 32 | 10 | 2.5 | positive | 7.07 |
| Muhallah Raja Sultan | Jan-15 | 12 | 67 | 32 | 10 | 2.5 | positive | 7.05 |
| DhokeNaju bridge | Jan-15 | 12 | 67 | 32 | 10 | 2.5 | positive | 7.35 |
| Nikki lai, | Jan-15 | 12 | 67 | 32 | 10 | 2.5 | negative | 7.13 |
| EidGah | Jan-15 | 12 | 67 | 32 | 10 | 2.5 | positive | 6.99 |
| National Market | Feb-15 | 14 | 66 | 50 | 11 | 4.5 | negative | 7.37 |
| Rawal lake | Feb-15 | 14 | 66 | 50 | 11 | 4.5 | positive | 7.48 |
| Banni Gala | Feb-15 | 14 | 66 | 50 | 11 | 4.5 | positive | 7.48 |
| G-10/4 | Apr-15 | 23 | 60 | 170 | 19 | 3 | negative | 7.56 |
| G-9/1 | Apr-15 | 23 | 60 | 170 | 19 | 3 | negative | 7.94 |
| G-9/4 | Apr-15 | 23 | 60 | 170 | 19 | 3 | negative | 7.7 |
| G-8/3 | Apr-15 | 23 | 60 | 170 | 19 | 3 | negative | 7.78 |
| G-8/1 | Apr-15 | 23 | 60 | 170 | 19 | 3 | negative | 8.2 |
| H-10 | May-15 | 29 | 52 | 32 | 22 | 7.5 | negative | 6.89 |
| H-9/4 | May-15 | 29 | 52 | 32 | 22 | 7.5 | negative | 8.02 |
| H-8/4 | May-15 | 29 | 52 | 32 | 22 | 7.5 | negative | 8.56 |
| H-8/1 | May-15 | 29 | 52 | 32 | 22 | 7.5 | negative | 8.7 |
| I-10/4 | May-15 | 29 | 52 | 32 | 22 | 7.5 | negative | 7.99 |
| I-8/1 | May-15 | 29 | 52 | 32 | 22 | 7.5 | negative | 8.96 |
| I-9 | May-15 | 29 | 52 | 32 | 22 | 7.5 | negative | 8.68 |
| New katarian bridge | May-15 | 29 | 52 | 32 | 22 | 7.5 | negative | 7.88 |
| Ameen Town | Jun-15 | 30 | 45 | 12.2 | 20 | 5 | negative | 7.55 |
| Muhallah Raja Sultan | Jun-15 | 30 | 45 | 12.2 | 20 | 5 | negative | 8.31 |
| DhokNaju bridge | Jun-15 | 30 | 45 | 12.2 | 20 | 5 | negative | 7.34 |
| Nikki Lai | Jun-15 | 30 | 45 | 12.2 | 20 | 5 | negative | 6.9 |
| EidGah | Apr-15 | 23 | 60 | 170 | 19 | 3 | negative | 7.84 |
| National Market | Apr-15 | 23 | 58 | 170 | 19 | 3 | negative | 6.99 |
| Rawal lake | Apr-15 | 23 | 60 | 170 | 19 | 3 | negative | 7.65 |
| Banni Gala | Apr-15 | 23 | 60 | 170 | 19 | 3 | negative | 7.96 |

**Supplementary Table 2:** Primer sequences used for nested PCR for Rotavirus detection

| **Primer Name** | **Sequence** |
| --- | --- |
| BEG9 | 5’GGC TTT AAA AGA GAG AAT TTC CGT CTG G3’ |
| END9 | 5’GGT CAC ATC ATA CAA TTC TAA TCT AAG3’ |
| VP7-F | 5'ATG TAT GGT ATT GAA TAT ACC AC 3' |
| VP7-R | 5' AAC TTG CCA CCATTT TTT CC 3' |
